# DISCO: Species Tree Inference using Multicopy Gene Family Tree Decomposition

**DOI:** 10.1093/sysbio/syab070

**Published:** 2021-08-27

**Authors:** James Willson, Mrinmoy Saha Roddur, Baqiao Liu, Paul Zaharias, Tandy Warnow

**Affiliations:** Department of Computer Science, University of Illinois at Urbana-Champaign, Urbana, IL 61801, USA; Department of Computer Science, University of Illinois at Urbana-Champaign, Urbana, IL 61801, USA; Department of Computer Science, University of Illinois at Urbana-Champaign, Urbana, IL 61801, USA; Department of Computer Science, University of Illinois at Urbana-Champaign, Urbana, IL 61801, USA; Department of Computer Science, University of Illinois at Urbana-Champaign, Urbana, IL 61801, USA; Department of Computer Science, University of Illinois at Urbana-Champaign, Urbana, IL 61801, USA

## Abstract

Species tree inference from gene family trees is a significant problem in computational biology. However, gene tree heterogeneity, which can be caused by several factors including gene duplication and loss, makes the estimation of species trees very challenging. While there have been several species tree estimation methods introduced in recent years to specifically address gene tree heterogeneity due to gene duplication and loss (such as DupTree, FastMulRFS, ASTRAL-Pro, and SpeciesRax), many incur high cost in terms of both running time and memory. We introduce a new approach, DISCO, that decomposes the multi-copy gene family trees into many single copy trees, which allows for methods previously designed for species tree inference in a single copy gene tree context to be used. We prove that using DISCO with ASTRAL (i.e., ASTRAL-DISCO) is statistically consistent under the GDL model, provided that ASTRAL-Pro correctly roots and tags each gene family tree. We evaluate DISCO paired with different methods for estimating species trees from single copy genes (e.g., ASTRAL, ASTRID, and IQ-TREE) under a wide range of model conditions, and establish that high accuracy can be obtained even when ASTRAL-Pro is not able to correctly roots and tags the gene family trees. We also compare results using MI, an alternative decomposition strategy from Yang Y. and Smith S.A. (2014), and find that DISCO provides better accuracy, most likely as a result of covering more of the gene family tree leafset in the output decomposition. [Concatenation analysis; gene duplication and loss; species tree inference; summary method.]

The estimation of species trees is a basic step in biological discovery, but it is challenged by gene tree heterogeneity (i.e., when there is a difference between the evolutionary trees of genes and species). These differences can be caused by a variety of factors, including incomplete lineage sorting (ILS), gene duplication and loss (GDL), and horizontal gene transfer (HGT).

Many methods have been developed to estimate species trees in the presence of ILS, with the major current approach (due to its computational tractability) being methods, such as ASTRAL (Mirarab et al. 2014), that operate by first inferring a tree for each gene family, then combining gene trees into a species tree based on some optimization criterion; many of these methods, which are called “summary methods” have been proven to be statistically consistent under the multispecies coalescent (MSC) model (Takahata, 1989; Hudson, 1983) (an extension of Kingman’s coalescent (Kingman, 1982) to the multi-species case); see Allman et al. (2016), Roch and Warnow (2015), Liu et al. (2010), and Larget et al. (2010) for an entry to this literature. Other approaches for species tree estimation in the presence of ILS include *BEAST (Heled and Drummond 2009), which is a Bayesian method that coestimates gene trees and the species tree from the multilocus set of gene sequence alignments, and SVDquartets (Chifman and Kubatko 2014), which takes as input a concatenation of the gene sequence alignments, estimates quartet trees (one tree for every four species) and then combines the quartet trees using a quartet amalgamation heuristic. Finally, another well-established approach is to concatenate the alignments of all the gene families and then infer a tree using techniques such as maximum likelihood (e.g., RAxML; Stamatakis 2014). However, these techniques require genes with at most one copy in each species, and so cannot be used directly in the presence of another primary source of heterogeneity—GDL.

The introduction of gene duplication and loss adds paralogs alongside orthologs, where paralogs are genes that have evolved from a common ancestor via a duplication event, whereas orthologs evolved from a speciation event (Fitch 2000; Yang and Smith 2014). Thus, gene families can contain multiple genes with the same species label. One way to address this problem is to first determine which gene copies are orthologs and then use this information in a subsequent analysis. However, while several techniques have been developed to improve orthology detection (surveyed in Altenhoff et al. (2019)), current methods can fail to correctly detect orthology under some conditions. As an example, reconciliation methods (which compare a provided gene tree to an established species tree) can have errors when either the gene tree or species tree have errors. More accurate methods exist that depend on external information (e.g., functional annotations and synteny), but can still make mistakes. In general, orthology detection is challenging and not yet considered solved.

Several methods have been developed to infer species trees from multicopy gene families without requiring orthology determination. For example, Boussau et al. (2013) presented Phyldog, a Bayesian method that coestimates the species tree and gene family trees, and De Oliveira Martins et al. (2016) presented *guenomu,* a Bayesian supertree method that combines gene family trees into a species tree. Other approaches that are better able to scale to large data sets have been developed, including iGTP (Chaudhary et al. 2010), DupTree (Wehe et al. 2008), DynaDup (Bayzid et al. 2013), STAG (Emms and Kelly 2018), and MulRF (Chaudhary et al. 2015b), as well as newer methods such as FastMulRFS (Molloy and Warnow 2020), ASTRAL-Pro (Zhang et al. 2020), SpeciesRax (Morel et al. 2021), and MiniNJ (Morel et al. 2021). These methods construct species trees by combining estimated gene family trees, some using parsimony-style approaches (e.g., minimizing the total number of duplications and losses). Finally, as studied in (Yan et al., 2022), species tree estimation can also be accomplished by treating multi-copy gene families as multi-individual inputs. For example, ASTRAL, which has an implementation (Rabiee et al. 2019) suitable to multi-allele data sets, can be used with multicopy gene family trees.

The theoretical properties of species tree estimation methods when GDL is present have not yet been fully established. However, ASTRAL-multi, which is designed to infer species trees from multi-individual trees (not multicopy trees) in the presence of ILS, was proven (Legried et al. 2021) to be statistically consistent under the (Arvestad et al. 2009) GDL model and subsequently also proven consistent (Markin and Eulenstein 2021) under the Rasmussen and Kellis (2012) DLCOAL model. ASTRAL-Pro, a variant of ASTRAL developed to explicitly address GDL, was proven consistent under the Arvestad *et al.* GDL model under the assumption that it correctly “roots and tags” each gene family tree (thus correctly identifying orthologs). FastMulRFS was also proven statistically consistent under fairly generic GDL models but only under the assumption that no “adversarial” GDL occurs, a property achieved under duplication-only or loss-only models (Molloy and Warnow 2020).

Of equal interest is the empirical performance of species tree estimation methods when paralogy is present. Many different approaches have been proposed that vary in terms of how multicopy gene families are handled in order to enable species tree estimation from gene family trees (Smith and Hahn 2021). Evaluation of these methods and preprocessing strategies on simulated data sets have generally shown encouraging results and suggested that relying on correct orthology detection is not necessary for highly accurate species trees (Yan et al. 2022). Several studies have nevertheless found differences between species tree estimation methods and preprocessing strategies. For example, Zhang et al. (2020), Molloy and Warnow (2020), and Legried et al. (2021) have shown that ASTRAL-multi (a method established to be statistically consistent under standard GDL models) is more accurate than DupTree and STAG but not as accurate as many other methods, including MulRF, FastMulRFS, ASTRAL-Pro, and ASTRID-multi. In addition, Chaudhary et al. (2015a) found NJst to be less accurate than gene tree parsimony and MulRF. Zhang et al. (2020), Willson et al. (2021), and Yan et al. (2022) have also shown that ASTRAL-Pro matches or improves on the accuracy of FastMulRFS and ASTRID-multi under conditions with GDL and ILS. Finally, Morel et al. (2021) has shown SpeciesRax and MiniNJ to be more accurate than ASTRAL-Pro under conditions with GDL, ILS, and HGT.

Another approach is to detect orthologs and discard paralogs by decomposing multicopy gene family trees into single copy trees. Several methods of this type have been proposed (Hejnol et al. 2009; Dunn et al. 2013; Kocot et al. 2013; Yang and Smith 2014; Ballesteros and Hormiga 2016; Thalén 2018). The PhyloPyPruner website (Thalén 2021) provides an implementation and review of many of these techniques.

Some of these decomposition techniques have been used in biological data sets and have been shown to be promising when combined with multilocus species tree estimation methods (e.g., see Yang and Smith 2014; Cheon et al. 2020). As found in Cheon et al. (2020), one of the most promising such techniques repeatedly finds and extracts a “maximum inclusive” subtree (i.e., the largest subtree possible without including two or more leaves labeled with the same species). Some of the variants of this approach require that the gene family tree be rooted (e.g., the “treeprune” script from the Agalma pipeline (Dunn et al. 2013) which is based on an approach from Hejnol et al. (2009) and PhyloTreePruner (Kocot et al. 2013). Some remove only one single copy tree per gene family tree while others iteratively remove the maximally inclusive subtrees and return all the extracted trees. The iterative use of the Maximum Inclusive approach, applied to unrooted gene family trees, is one of the techniques provided in Yang and Smith (2014), where it is referred to as “MI.” However, these decomposition strategies have not been explored on simulated data sets; hence, their relative performance is not yet fully evaluated.

Here, we introduce Decomposition Into Single COpy gene trees (DISCO), a new method for decomposing multicopy gene family trees that takes advantage of ASTRAL-Pro’s “rooting and tagging” algorithm. Our method decomposes the input in such a way as to prioritize creating at least one large single copy tree, while also retaining single copy subtrees that are split off. We prove that ASTRAL-DISCO, the combination of DISCO with ASTRAL, is statistically consistent under GDL models provided that ASTRAL-Pro correctly roots and tags each gene family tree. We compare DISCO to MI, another decomposition strategy (Yang and Smith 2014) and show that pairing ASTRAL with DISCO instead of MI produces more accurate species trees, and that DISCO’s decomposition strategy covers more of the input gene family tree leafset than MI’s. We find through a simulation study that the DISCO decomposition paired with ASTRID (a pipeline that we refer to as ASTRID-DISCO) performs surprisingly well, generally performing the best in cases where the gene trees have acceptable accuracy. Another technique we examine is the use of concatenation analysis enabled using our decomposition approach (CA-DISCO); this method gives the species tree with the best accuracy in many cases, especially in instances where the initial gene trees have low accuracy. We also test our methods on several published empirical data sets (some of which are too large for CA-DISCO) and find that ASTRID-DISCO produces reasonable results, agreeing with reference trees for established clades, and comparable to ASTRAL-Pro in terms of accuracy while being much faster.

## Materials and Methods

### Multicopy Gene Tree Decomposition

When a duplication event occurs, the gene is copied to another locus in the genome, and after that, genes both from the parent locus and the child locus are subject to further duplication and loss events as well as ILS. As a multicopy gene tree accounts for the evolutionary history (including duplication events) of all the copies of a specific gene over the whole taxon set, its leaves are spread across multiple loci. The subtrees rooted at the children of each duplication node correspond to the evolutionary history of the gene on different loci. We note also that some models of gene duplication and loss make the simplifying assumption that the gene copies evolve independently.

**Figure 1.**
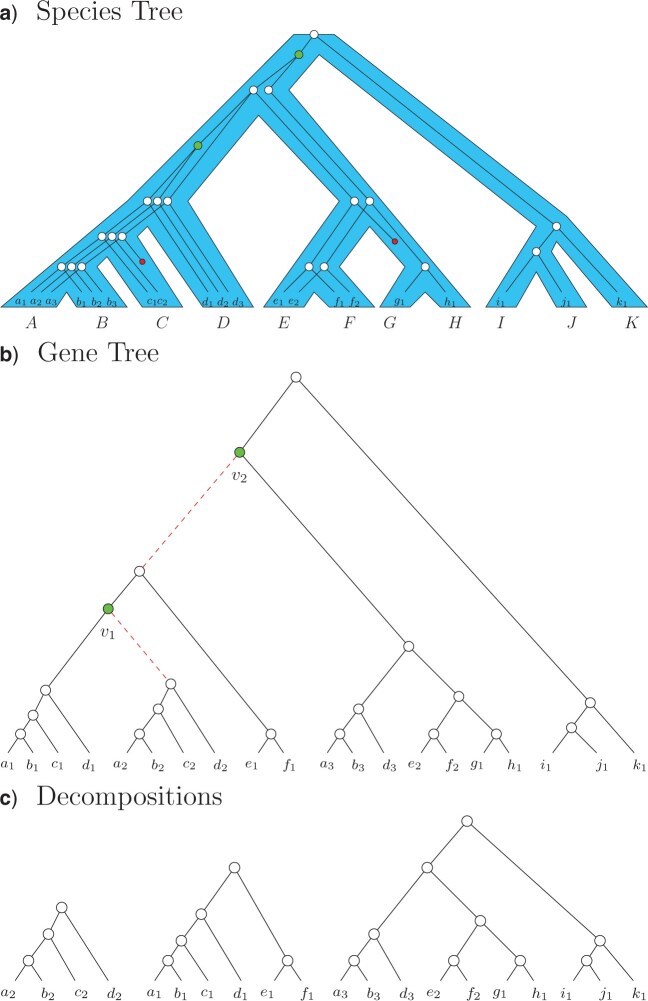
Decomposition algorithm. In a), the gene tree is shown inside the species tree. White nodes (open circles in print) correspond to speciation events and green nodes (filled-in nodes in print) denote duplication events. The genes $a_i, b_i, \ldots, k_i$ belong to species $A, B, \ldots, K$ respectively. The resulting multicopy gene tree is shown in (b). The decomposition method deletes the child edge corresponding to the smallest subtree for each duplication node and outputs subtrees generated after the edge deletion process. This is done postorder (bottom up). In our example b), we have two duplication vertices. First, we examine the duplication vertex closest to the bottom of the tree, $v_1$; it has two child clades, both containing four species, so we arbitrarily split off the right one. When we examine the next duplication vertex, $v_2$, we now have six species in the left subtree and seven in the right subtree, so we disconnect the left one. The resulting decomposition is shown in (c), which is the result of deleting the dashed edges in (b).

To run summary methods designed for single copy gene family trees such as ASTRID and ASTRAL, we introduce a method for decomposing these multicopy gene family trees into single copy trees. The input of the decomposition algorithm is a set of multicopy, unrooted, gene family trees, and for each tree the method produces multiple single copy trees. The decomposition algorithm attempts to break each multicopy gene tree spreading across different loci into smaller subtrees containing only orthologs (Fig. 1). The method works in two steps as follows:

In the first step, we root the gene trees then label the internal vertices of the tree as either duplication events or speciation events (a process called “tagging”). To accomplish this we use ASTRAL-Pro’s rooting and tagging algorithm (Zhang et al. 2020). The algorithm scores each edge in the gene tree based on the minimum number of duplication or loss events that would be required to explain a tree rooted on that edge, then roots the tree on the edge with the minimum score. Once rooted, the internal vertices that are the least common ancestor of two copies of the same species are considered to be duplication events. For example, suppose internal node $v$ has species $A$ as a leaf in both the right subtree and the left subtree; then $v$ is labelled (“tagged”) as a duplication event. For this step, the gene trees are assumed to be binary (if the input contains polytomies, they are randomly resolved).

The second step is to decompose the rooted and tagged gene family trees (Algorithm 1). We visit each vertex with postorder traversal (this means we visit child vertices before their parents, traversing the tree from the bottom up); when we reach a vertex tagged as a duplication, we resolve it by separating one of the two child clades below the vertex (we choose the one containing the smallest number of species).

Algorithm 1 is guaranteed to decompose the input gene family tree into leaf-disjoint subset trees, each of which is single copy. This set of single copy trees will contain one (possibly large) tree as well as all the clades separated during the decomposition. Furthermore, assuming correct rooting and tagging, these subtrees only contain orthologs as all duplication vertices have been eliminated.

This set of single copy gene trees can then be given as input to a summary method, such as ASTRAL or ASTRID, thus producing a pipeline we refer to as ASTRAL-DISCO or ASTRID-DISCO, respectively. To use this pipeline with concatenation, we make the following modification. We follow the first two steps (i.e., using ASTRAL-Pro to root and tag the gene family trees, and then decompose the gene family trees into single copy gene trees). Then, we separate the sequences for each gene family based on the decomposition, placing the sequences corresponding to leaves in each decomposed tree into separate sets of sequences. These sequence alignments are then concatenated into a large supermatrix, and the desired concatenation analysis method can be run to obtain the species tree. For our study we used the maximum-likelihood method IQ-TREE (Nguyen et al. 2015), and refer to this pipeline as concatenation analysis with DISCO (CA-DISCO).

**Algorithm 1:**
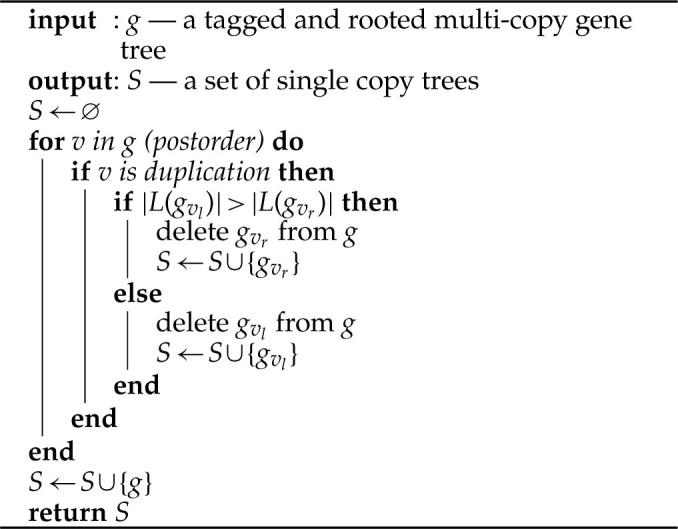
disco Step 2 *NOTES:* If $t$ is a tree, then we define $L(t)$ to be leafset of the tree. Also given a vertex from $t$, say $v$, we define $t_{v_l}$ and $t_{v_r}$ to be the left and right subtrees of vertex $v$ respectively. We delete a subtree from a tree by removing the edge above the root of the subtree. We obtain the tagged and rooted multicopy gene tree used as the input by running the ASTRAL-Pro rooting and tagging algorithm.

#### Statistical consistency properties of ASTRAL-DISCO

Here, we establish statistical consistency for ASTRAL-DISCO under GDL-only models, and compare this statistical consistency guarantee to what has been established for ASTRAL-Pro in (Zhang et al., 2020).

Theorem 1. Under a model of gene evolution in which the only cause for gene tree heterogeneity is GDL and the true species tree has strictly positive probability of appearing as a gene tree, ASTRAL-DISCO is statistically consistent provided that ASTRAL-Pro correctly roots and tags the gene family trees.

Proof.
We let $T$ denote the unrooted species tree topology, let $f$ be one of the gene family trees, and let $t$ be any DISCO tree produced by applying DISCO to $f$ (so that $t$ is one of the trees in the decomposition of $f$). Under the assumption that ASTRAL-Pro has correctly rooted and tagged $f$, the leaves in $t$ are all orthologs and hence the species tree $T$ will induce the subtree $t$ when restricted to the leaves of $t$. Therefore, $T$ will be a compatibility supertree of the DISCO trees. Furthermore, when restricting to a set of four leaves $a,b,c,d$ in $t$, the species tree $T$ and the DISCO tree $t$ will induce the same quartet tree topology. Therefore, the true species tree $T$ will have the maximum possible score with respect to the Maximum Quartet Support Species Tree (MQSST) problem (Mirarab et al. 2014) on the decomposed DISCO trees. As the number of genes goes to infinity, almost surely a gene tree topologically identical to the species tree topology $T$ will appear in the input. With $T$ in the input, the *only* optimal solution to the MQSST problem on the decomposed trees is the species tree, as any other solution will disagree with $T$ in at least one quartet. Furthermore, with $T$ in the input, the species tree will also be a feasible solution to the constrained-MQSST problem solved by ASTRAL-DISCO as the bipartitions of $T$ will appear in the constraint set computed by ASTRAL (which always includes all the bipartitions from the input gene trees). Hence, under the conditions of the theorem, for every model species tree, as the number of genes increases, almost surely ASTRAL-DISCO will return the unrooted species tree topology as its unique solution. □

*Comments:* There are two specific requirements on the gene evolution model stated in the theorem: i) the only cause for discord between the topologies of the unrooted gene trees and the species tree is GDL and ii) the unrooted topology of the true species tree has strictly positive probability of appearing as a gene tree. These two properties hold for the Arvestad et al. (2009) GDL model. Furthermore, these conditions are very mild and so will hold for other GDL models as well.

We now consider the requirement that ASTRAL-Pro correctly roots and tags every gene family tree. This requirement, which is also stated in the theorem in Zhang et al. (2020) for ASTRAL-Pro’s statistical consistency under GDL, is not likely to hold on many conditions, since gene family trees that are single copy but not identical to the species tree will occur with strictly positive probability, and ASTRAL-Pro will not consider any node in such a tree to be a duplication node. Hence, ASTRAL-Pro is not able to correctly root and tag such gene family trees. Thus, this requirement is nontrivial, and far more restrictive than the two requirements on the GDL model.

We now compare the theoretical guarantees established here for ASTRAL-DISCO, and compare these guarantees to those established for ASTRAL-Pro. The conditions required in (Zhang et al., 2020) under which ASTRAL-Pro is statistically consistent are the same as the ones we state in Theorem 1 under which ASTRAL-DISCO is statistically consistent: GDL is the only cause for gene tree discord, the true species tree has strictly positive probability of appearing as a gene tree, and ASTRAL-Pro correctly roots and tags every gene family tree. Hence, the two methods have the same theoretical guarantee.

Although the result is trivially true, (Zhang et al., 2020) also note that ASTRAL-Pro is statistically consistent under the multispecies coalescent (MSC) model (which addresses incomplete lineage sorting) and the same guarantee holds for ASTRAL-DISCO since in that case the ASTRAL-DISCO is identical to ASTRAL. (Zhang et al., 2020) also specifically discuss the question of ASTRAL-Pro consistency under models where gene duplication and loss as well as incomplete lineage sorting occur. They conjecture that ASTRAL-Pro might be consistent under such models (provided, of course, that ASTRAL-Pro correctly root and tag each gene family tree), but have not yet been able to establish this property.

In sum, to date there are no statistical consistency guarantees established for ASTRAL-Pro that do not also hold for ASTRAL-DISCO. Both methods are proven statistically consistent when GDL is the only cause for gene tree discord (provided correct rooting and tagging) and unknown statistical consistency for other models. The dependency of all these proofs on correct rooting and tagging is a significant one, but it is possible that with random but low probability of error in rooting and tagging that each method will be proven statistically consistent under a GDL-only model. The lack of distinction between the two methods with respect to theoretical guarantees means that the empirical performance must be evaluated, which is the subject of the next section.

### Experiment Overview

Full details (including software version numbers and commands necessary to reproduce the experiment) are provided in Sections S1 and S2 of the Supplementary material. Data sets used in this study are available from the Illinois Databank at https://databank.illinois.edu/datasets/IDB-4050038, and the DISCO algorithm is freely available at https://github.com/JSdoubleL/DISCO. Here, we provide an overview of the experimental design, which included three main experiments.

Experiment 1 has two parts. In Experiment 1(a), we compare DISCO and MI to evaluate the relative benefits of these two decomposition strategies. This experiment shows that DISCO provides better accuracy than MI, and covers more of the input gene family tree leafset in its decomposed subset trees. Experiment 1(b) provides an initial evaluation of a collection of species tree estimation methods, some using DISCO with ASTRAL or ASTRID, on a number of different simulation model conditions. This experiment provides us with a list of species tree estimation methods we then explore more fully in the next experiments. In Experiment 2, we explore a larger number of simulated model conditions, and we include a comparison to CA-DISCO. Finally, in Experiment 3, we examine the most accurate and scalable methods on three empirical data sets of increasing size.

For all the experiments, we compare the species tree estimation methods with respect to their topological error (measured by the normalized Robinson–Foulds [RF] distance (Robinson and Foulds 1981) between the true or reference species tree and the species tree returned by the method) as well as the wall-clock running time (starting at the beginning of each pipeline or method and ending once the species tree is obtained; time spent estimating the gene family trees is not included). For Experiments 2 and 3, we also record the peak memory usage of the respective methods. Finally, in comparing MI and DISCO, we report the coverage of the decomposed trees, which is the proportion of the number of leaves in the full set of decomposed trees out of the original gene family tree.

For each experiment, we compare methods only on those replicate data sets on which all the methods complete analyses. We omit a method from a figure if it fails to complete on 50% or more of the replicates; these cases are reported in each figure.

We run our experiments on the Campus Cluster at the University of Illinois Urbana-Champaign. The Campus Cluster has a four-hour time limit on jobs and is heterogeneous, with a minimum of 64 GB of memory. Additionally, for Experiment 3, we use the Tallis queue, which has 256 GB of available memory and effectively no time constraints.

### Methods Compared

DISCO creates a set of single copy gene trees from the input set of gene family trees. Thus, we can pair DISCO with any existing multilocus species tree estimation method, including ASTRID, ASTRAL, and concatenation analysis (CA). Here, we compare ASTRID-DISCO, ASTRAL-DISCO, and CA-DISCO to existing methods that can analyze multicopy gene family trees. Note that CA-DISCO works from the concatenation of the multiple sequence alignments rather than the gene trees.

In addition to these combinations of DISCO with ASTRAL, ASTRID, and CA, we examine the performance of the following leading methods for species tree inference under gene duplication and loss.

#### FastMulRFS

FastMulRFS is an extension of FastRFS (Vachaspati and Warnow, 2017) that allows it to work for multicopy gene family trees. It accomplishes this by adding a preprocessing step that compresses multicopy gene family trees into single copy trees by collapsing any edge in the tree separating two leaves with the same label. In Molloy and Warnow (2020), it was shown to be statistically consistent under a model of gene duplication and loss that assumes a lack of *adversarial GDL* (this is when specific sequences of gene duplication and loss events create bipartitions that conflict with the species tree). FastMulRFS was shown to exhibit equivalent accuracy to MulRF and to be more accurate than ASTRAL-multi in Molloy and Warnow (2020); it also significantly outperformed both methods in terms of speed.

#### ASTRAL-Pro

ASTRAL-Pro is an extension of ASTRAL designed for multi-copy gene trees. ASTRAL-Pro uses a multistep process: first, it roots and tags the gene trees, selecting a root that minimizes the number of duplication and loss events necessary to explain the tree, and then it runs standard ASTRAL, but only counting quartets where the least common ancestor (LCA) for any three out of the four leaves is a speciation vertex and the four leaves of the quartet all refer to different species (also these “speciation-driven quartets” are counted as equivalent if they cover the same four species and share the same LCA; for more details see Zhang et al. (2020). In Zhang et al. (2020), ASTRAL-Pro was shown to be statistically consistent under a model of gene duplication and loss with the added assumption that the rooting and tagging is correct; it was also shown to be more accurate and faster than MulRF, DupTree, and ASTRAL-multi. Further, we found that ASTRAL-Pro outperformed FastMulRFS and ASTRID-multi in Willson et al. (2021).

#### ASTRID-multi

ASTRID-multi is an extension of ASTRID (Vachaspati and Warnow, 2015). ASTRID creates a distance matrix with the average inter-node distance between every pair of species over all the input trees. This matrix is then used as the input to a distance-based method that can produce a tree (by default it uses FastME; Lefort et al. 2015). ASTRID-multi allows for multi-individual trees using a technique from Allman et al. (2016); it was shown to be competitive with other methods for inferring species trees with gene duplication and loss in Legried et al. (2021), where it performed better than STAG, DupTree, ASTRAL-multi, and MulRF.

#### SpeciesRax and MiniNJ

SpeciesRax (Morel et al. 2021) is a new maximum likelihood-based species tree inference method. The program begins by generating a starting species tree; from that tree, it begins a search for a tree that maximizes the probability of observing the input trees under the *undatedDTL* model (Morel et al. 2020). The starting tree can be randomly generated or computed from the input trees using MiniNJ, also introduced in Morel et al. (2021). MiniNJ uses internode distances like ASTRID; however, when building its distance matrix, it takes the minimum distance between each pair of species averaged across all the gene family trees, then from the distance matrix it estimates the tree using Neighbor Joining (Saitou and Nei 1987).

### Data Sets

We use both simulated and biological data sets to evaluate methods; high-level descriptions of these data sets are provided here; see Section S3 of the Supplementary material for additional empirical statistics.

For our simulation study, we use SimPhy (Mallo et al. 2016) to generate species trees and true gene trees under the DLCOAL model (Rasmussen and Kellis 2012), a model including both ILS and GDL; we ran SimPhy with parameters which avoid a strict molecular clock. SimPhy first evolves a locus tree within the species tree with gene duplications and losses, and then evolves the true gene family tree within the locus tree under the MSC model. Thus, the locus tree differs from the species tree due to GDL but not ILS, and the true gene family tree differs from the locus tree due to ILS. We then use INDELible (Fletcher and Yang 2009) to simulate sequence evolution (with different sequence lengths) under the GTR (Tavaré 1986) model of sequence evolution with gamma distributed rates (Table S1 of the Supplementary material). From these sequence alignments, we estimate gene trees using FastTree2 (Price et al. 2010). We simulate these data sets under various model conditions to produce a range of data sets that vary in terms of ILS, GDL, average gene family size, number of species per gene family tree, and mean gene tree estimation error (MGTE); see Tables S2 and S3 for these properties. The MGTE is the average Robinson–Foulds (RF) distance between the estimated gene family tree and the true gene family tree (Robinson and Foulds 1981) divided by $2n-6$, where $n$ is the number of leaves in the gene family tree. The ILS level is reported using the average distance (AD), computed using RF distances between the locus tree (which has no ILS) and the true gene family tree, divided by $2n-6$. When computing the RF distance between two multi-copy trees, unique leaf labels are used for every copy of a species.

#### Default Conditions

Our default values for the parameters not actively being examined are: 101 species (100 in-group species and one outgroup species), 50 genes trees estimated from 100bp alignments (43% MGTE), a moderate level of ILS using a haploid effective population size of $5.0 \times 10^7$ (20% AD), and a moderate duplication rate of $5.0 \times 10^{-10}$ with an equal loss rate.

#### Varying MGTE

Keeping the default parameter values otherwise fixed, we vary the mean gene tree estimation error (MGTE) by creating sequences of different lengths with INDELible. We create datasets with sequences 500 bp, 100 bp, and 50 bp long, which correspond to 19%, 43%, and 56% MGTE, respectively.

#### Varying ILS

Keeping the default parameter values otherwise fixed, we changed the amount of discord due to ILS by varying the haploid effective population size, choosing values of $1.0 \times 10^{4}$, $5.0 \times 10^{7}$, and $2.0 \times 10^{8}$, which correspond to an average distance (AD—the normalized RF distance between the true species trees and the true gene trees) of 0%, 20%, and 50% respectively (higher precision AD values are provided in Table S2 of the Supplementary material).

#### Varying GDL

Keeping the default parameter values otherwise fixed, we also varied the amount of gene duplication as well as the relative probability of gene loss. We chose duplication rates of $1.0 \times 10^{-10}$ (low), $5.0 \times 10^{-10}$ (moderate), and $1.0 \times 10^{-9}$ (high) as well as relative loss rates of 0, 0.5, and 1 times the duplication rate, for a total of nine model conditions. This resulted in trees that varied significantly in size (number of leaves), as shown in Table 1.

**Table 1.** Average number of leaves in the true gene family trees for model conditions with 101 species and differing amounts of gene duplication and loss. Results shown are averaged across 1000 genes and 10 replicates each.

	L/D=0	L/D=0.5	L/D=1
$1\times 10^{-10}$	145.1	128.0	116.6
$5\times 10^{-10}$	550.0	290.6	165.3
$1\times 10^{-9}$	3727.8	993.0	228.5

#### Varying number of genes and missing data

Keeping the default parameter values otherwise fixed, we created data sets with 10, 50, 100, 500, 1000, and 10,000 genes, some also with “clade-based” missing data (Nute et al. 2018). For $\sim$60% of the gene family trees, a random clade containing 20% or more of the species was selected from the species tree; all the species not contained in this clade were then deleted from the gene tree. This way of deleting species is equivalent to having the selected gene being born below the root of the species tree. This process removed an average of 41.8% of the species from each of the selected gene family trees.

#### Large number of species

Keeping the default parameter values otherwise fixed, we also created a simulated data set with 1001 species (1000 in-group species and one outgroup) in order to test the scalability of the methods.

#### Empirical data sets

For Experiment 3, we examine three empirical data sets: (1) a 16-species fungi data set from Rasmussen and Kellis (2012), (2) the plant data set studied in Zhang et al. (2020) containing 9237 multi-copy gene trees and 83 species, and 3) an 188-species vertebrates data set with 31,612 gene families taken from (Morel et al. 2021) (who obtained their data from the NCBI Taxonomy Database; Federhen 2012). Each of these data sets is provided with estimated trees for each of the gene families. In addition, Rasmussen and Kellis (2012) and Morel et al. (2021) each provide a reference tree for their data set. For the reference tree on the plant dataset, we use the ASTRAL analysis of a larger plant data set from Leebens-Mack et al. (2019) to take advantage of denser taxon sampling used in Leebens-Mack et al. (2019) compared to Zhang et al. (2020). These reference trees provide a framework for evaluating the estimated species trees, but cannot be considered completely correct since some of the evolutionary relationships for these groups are still under debate. Details about the data sets and reference trees are available in the Supplementary material.

## Results

### Experiment 1

In Experiment 1, we have two separate experiments: 1(a) compares DISCO to MI and 1(b) evaluates different species tree estimation methods (some using DISCO). Both these experiments are based on simulated data sets.

#### Experiment 1(a): Comparing DISCO and MI

Here, we compare two tree decomposition strategies, DISCO and MI, on simulated data sets that evolve under one GDL model condition and with varying number of genes.

We first report statistics regarding DISCO decompositions, including average and maximum size (number of leaves) of the DISCO trees, average coverage of the gene family tree leafset, and number of DISCO trees across the different model conditions; see Table 2, Tables S4 and S5 and Figures S1 and S2 of the Supplementary material. These analyses show that the largest DISCO tree contains on average 79% of the species in the average gene family trees. These analyses also show that DISCO also produces many small trees and that the average size of a DISCO tree is under 8. (However, when DISCO is paired with summary methods, such as ASTRAL or ASTRID, DISCO trees with 3 or fewer leaves are discarded, which increases the average size of the DISCO tree to between 13 and 50, depending on the duplication rate.) Furthermore, after removing subset trees with fewer than 4 leaves, DISCO achieves coverage of 85%. In comparison, MI has a much smaller maximum tree size (17.3% compared to 57.9%) and lower coverage (62% compared to 85%).

**Figure 2.**
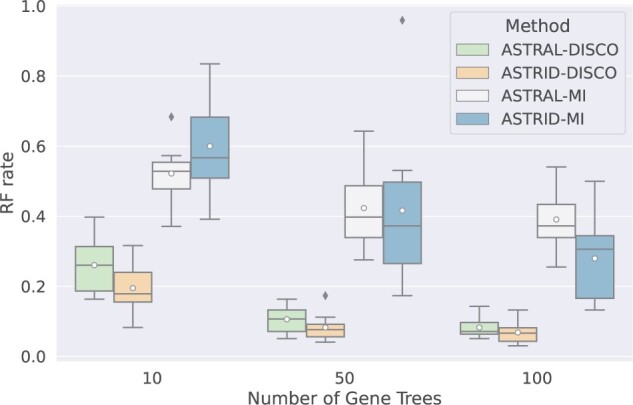
Comparison of the topological error of the species trees produced by ASTRID and ASTRAL paired with either DISCO or MI. All model conditions have 10 replicates with 101 species, 20% AD, 43% MGTE, and a duplication rate of $5 \times 10^{-10}$ with an equal loss rate.

We now evaluate species tree estimation using ASTRAL or ASTRID applied to the single copy gene trees produced by these two decompositions. As seen in Figure 2, using the DISCO decomposition instead of the MI decomposition produces much more accurate species trees for both ASTRAL and ASTRID. To understand these trends, we explore the decomposition statistics directly between DISCO and MI. As seen in Table 2, DISCO produces larger subset trees than MI and covers more of the input leafset across the gene family trees. Thus, there is more information in the set of single copy gene trees given to ASTRAL or ASTRID when using DISCO decompositions compared to using MI decompositions.

**Table 2.** Maximum tree size and coverage (proportion of leaves in the gene family tree that are contained in the decomposition method output set) produced by DISCO and MI, averaged across the gene family trees after removing the gene family trees with fewer than 4 species. For context, the average number of species in the gene family trees is 73.1, so that on average the largest DISCO tree has 79% of the species and the largest MI tree has 24% of the species. The model conditions have 101 species, 100 genes per replicate, 20% AD, 43% MGTE, and a duplication rate of $5 \times 10^{-10}$ with an equal loss rate.

Method	Maximum Size Tree	Coverage
MI	17.3	0.62
DISCO	57.9	0.85

#### Experiment 1(b): Varying numbers of gene trees

**Figure 3.**
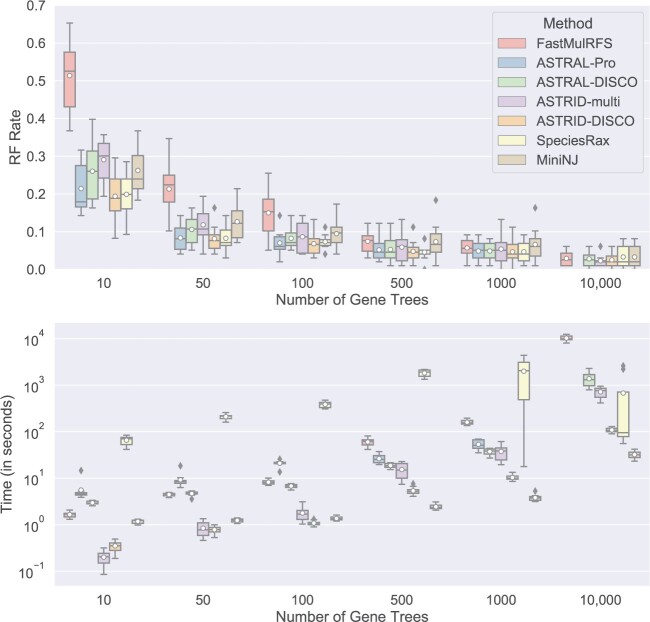
Experiment 1(b). Impact of number of genes on species tree error (RF error rates) and wall clock running time (seconds); results across 10 replicates per model condition are shown. All the data sets have 101 species (100 in-group species and one outgroup), gene trees estimated from 100bp alignments (43.3% MGTE), AD = 20%, a duplication rate of $5.0 \times 10^{-10}$ and an equal loss rate. ASTRAL-Pro failed to complete on 6/10 replicates for the 10,000-gene replicates, so it was omitted; FastMulRFS also failed on two replicates; results shown here are for the eight replicates on which FastMulRFS completed. All other methods completed on every replicate. The boxes stretch from the 1st to 3rd quartile and the lines through the boxes show the medians and the dots show means.

In our first experiment we examine summary methods that can estimate trees from multicopy gene family trees—FastMulRFS, ASTRAL-Pro, ASTRAL-DISCO, ASTRID-multi, ASTRID-DISCO, SpeciesRax, and MiniNJ—and explore performance (both accuracy and running time) on data sets with varying numbers of gene trees (Fig. 3). Given high numbers of gene trees, most methods have about the same accuracy; however, as the number of gene trees decreases, the differences between the methods become more pronounced. Notably, FastMulRFS, ASTRID-multi, and MiniNJ seem to degrade the most (with FastMulRFS exhibiting exceptionally poor accuracy when given 100 or fewer gene trees), while ASTRAL-Pro, ASTRID-DISCO, and SpeciesRax perform well. ASTRAL-DISCO performs well, but is not quite as accurate as ASTRAL-Pro. In contrast, ASTRID-DISCO distinctly improves on ASTRID-multi. The running times for all methods seem to increase with the number of genes at the same rate, with distance-matrix methods running the fastest and SpeciesRax running the slowest.

#### Experiment 1(b): varying gene duplication and loss rates

**Figure 4.**
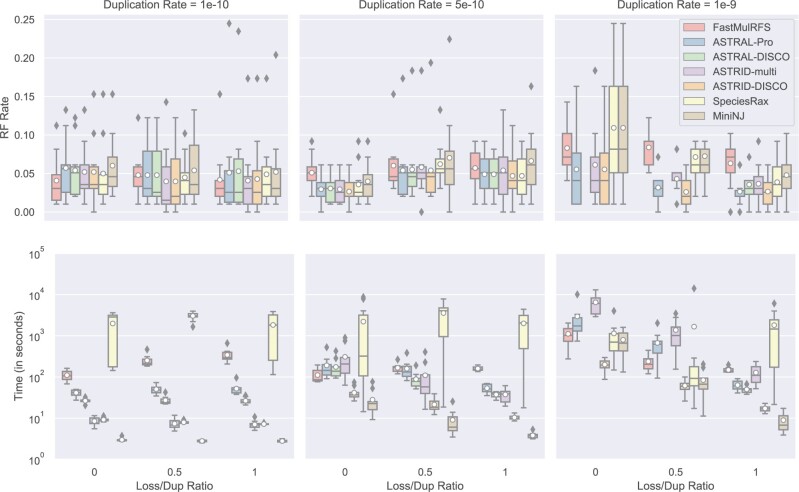
Experiment 1(b). Impact of GDL rate and loss/dup rate on species tree error (RF error rates) and wall clock running time (seconds); results across 10 replicates per model condition are shown. All the data sets have 101 species (100 in-group species and one outgroup), 1000 gene trees, gene trees estimated from 100 bp alignments (43.3% MGTE), and AD=20%. ASTRAL-DISCO failed on 90% of the replicates with loss = 0 and loss = 0.5 for duplication rate = 1e$-$9 due to memory; therefore, results for ASTRAL-DISCO are not shown for these two conditions. ASTRAL-Pro failed on two replicates and SpeciesRax failed on one (all from the model condition with the highest duplication rate and no loss); results shown here are for the replicates on which these methods completed.

We compare methods when running them on our nine data sets that vary gene duplication rates as well as the relative probability of gene loss (Fig. 4). The highest duplication rate shows the most difference between the methods, with SpeciesRax, MiniNJ, and FastMulRFS performing noticeably worse than the other methods. The running times reveal differences. ASTRID-multi is the slowest method at the highest duplication rate; ASTRID-DISCO and MiniNJ are the fastest by a large margin.

### Experiment 2

In this experiment, we continue to compare summary methods; however, we exclude summary methods that performed poorly in the previous experiment, limiting our focus to ASTRAL-Pro, ASTRID-DISCO, and SpeciesRax. We also include a comparison with CA-DISCO.

#### Varying numbers of gene trees

**Figure 5.**
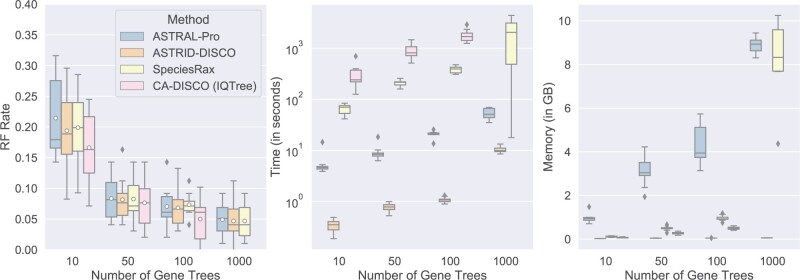
Experiment 2. Impact of number of genes on species tree error (RF error rates) and wall clock running time (seconds); peak memory usage is also given in gigabytes. Results across 10 replicates per model condition are shown. All the data sets have 101 species (100 in-group species and one outgroup), gene trees estimated from 100 bp alignments (43.3% MGTE), AD = 20%, a duplication rate of $5.0 \times 10^{-10}$ and an equal loss rate. CA-DISCO (IQ-TREE) timed out on all ten of the 1000-gene replicates, and so CA-DISCO is omitted from the 1000-gene results shown here.

First we extend our results from Experiment 1 that explored data sets with differing numbers of gene trees (Fig. 3) to include CA-DISCO and an examination of the methods’ peak memory usage (Fig. 5). CA-DISCO seems to have slightly better accuracy under most conditions; however, it fails to complete under our time constraints (4 h) with 1000 gene trees. CA-DISCO is also the slowest method. In most cases, ASTRAL-Pro is the least memory efficient; however, with 1000 gene trees, SpeciesRax exhibits high memory usage as well. The memory usage trends on the other conditions are very similar to the trends for this experiment, and are reported in Figures S3–S5 of the Supplementary material.

#### Varying GTEE

**Figure 6.**
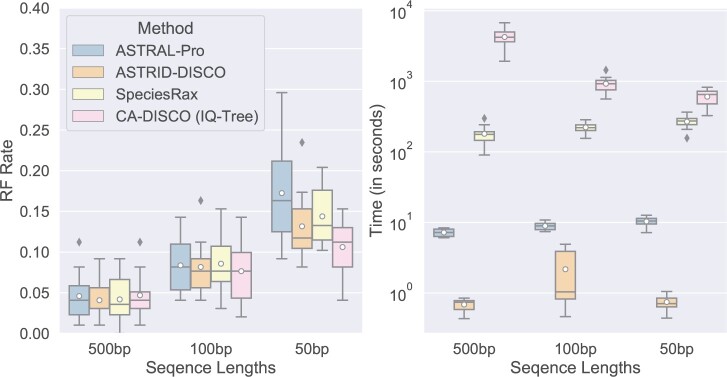
Experiment 2. Impact of mean gene tree estimation error (MGTE) on species tree error (RF error rates) and wall clock running time (seconds); peak memory usage is also given in gigabytes; results across 10 replicates per model condition are shown. All the data sets have 101 species (100 in-group species and one outgroup), 50 gene trees, AD = 20%, a duplication rate of $5.0 \times 10^{-10}$ and an equal loss rate. The data sets include gene trees estimated from three sequence lengths, and have MGTE of 19%, 43%, and 56%, respectively.

We run our methods on data sets with gene trees estimated from sequences of varying lengths (Fig. 6). Shorter sequences provide less information for estimating the gene trees, leading to gene trees with more estimation error; conversely longer sequences lead to less error. All methods have similar accuracy on long (500 bp) sequences, while CA-DISCO (closely followed by ASTRID-DISCO) performed the best out of the three methods on the data set with the shortest sequence lengths (50 bp). Running times remained fairly low for all methods summary methods; however, CA-DISCO uses a large amount of time, especially with longer sequences. ASTRAL-Pro and SpeciesRax take longer as sequence lengths decrease, while ASTRID-DISCO exhibits similar running times on all sequence lengths.

#### Varying ILS

**Figure 7.**
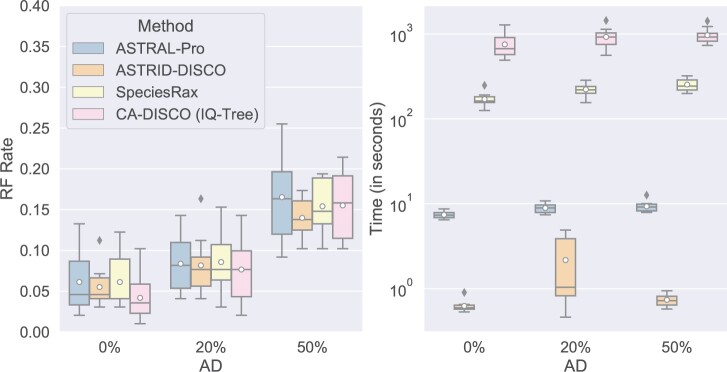
Experiment 2. Impact of ILS level on tree error (RF error rates), wall clock running time (s), and peak memory usage (gigabytes); results across 10 replicates per model condition are shown. All the data sets have 100 species, 50 gene trees estimated from 100 bp alignments (43.3% MGTE), a duplication rate of $5.0 \times 10^{-10}$, and an equal loss rate.

CA-DISCO exhibits better accuracy than the other three methods with no ILS (Fig. 7); however, as the ILS increases, ASTRID-DISCO shows the best accuracy when CA-DISCO loses its advantage, taking much more time than all other methods and delivering equivalent results to SpeciesRax and ASTRAL-Pro. Both SpeciesRax and CA-DISCO take a long time to complete, while ASTRID-DISCO is the fastest.

#### Varying duplication rate

**Figure 8.**
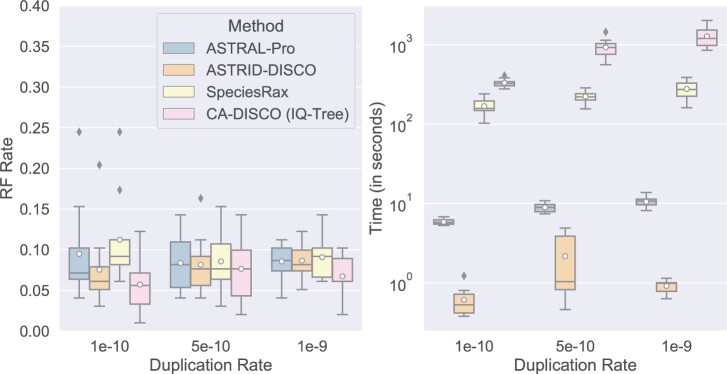
Experiment 2. Impact of duplication rate on species tree error (RF error rates), wall clock running time (s), and peak memory usage (gigabytes) on data sets with 50 genes; results across 10 replicates per model condition are shown. All the data sets have 101 species (100 in-group species and one outgroup), gene trees estimated from 100 bp alignments (43.3% MGTE), and AD = 20%, and an equal loss rate to the duplication rate.

Varying the duplication rate has little impact on any of the methods’ accuracy with only 50 genes (Fig. 8). At the lowest duplication rates both of the decomposition-based methods (CA-DISCO and ASTRID-DISCO) give trees with the best accuracy; as the duplication rates increase the differences fade, leaving CA-DISCO with only slightly better accuracy. Notably, with only 50 genes, SpeciesRaxâŁ™s accuracy is much more competitive with other methods than it was under the similar high duplication rate conditions with 1000 genes (Fig. 4). Running times exhibit similar trends to previous figures: ASTRID-DISCO is the fastest and SpeciesRax and CA-DISCO are the slowest.

#### Clade-based missing data

**Figure 9.**
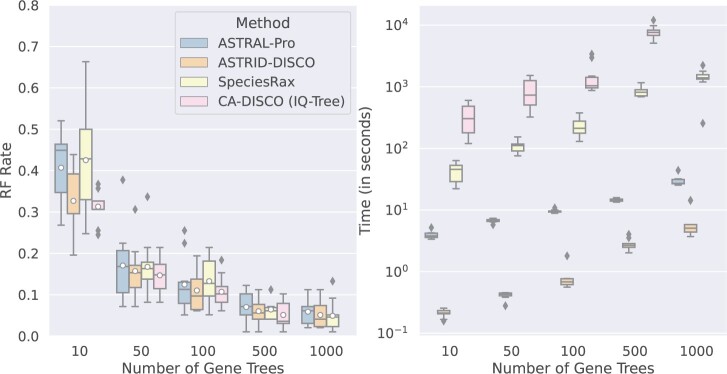
Experiment 2. Impact of number of gene trees on species tree error (RF error rates) and wall clock running time (seconds) under the missing data condition. For selected gene family trees, we selected a random clade containing at least 20% of the taxa from the species tree; species not found in this clade were deleted from the gene family tree. All data sets contain 101 species (100 in-group species and one outgroup) and gene trees estimated from 100bp alignments (43.3% MGTE). All the data sets have AD=20%, a duplication rate of $5.0 \times 10^{-10}$, an equal loss rate, and 10 replicates for each condition. CA-DISCO exceeded our four-hour time limit on 6 out of 10 of the 1000-gene replicates but completed on all replicates under all other conditions; hence, we removed CA-DISCO from the results shown for the 1000-gene replicates. All other methods complete on all replicates. Results shown here are averaged across only those replicates where all methods complete.

On our data sets with missing species (Fig. 9), we see that ASTRID-DISCO and CA-DISCO have much better accuracy with only 10 genes than other methods. As the number of genes increases, differences in accuracy between methods become less noticeable. The methods’ running times continue to exhibit the same trends.

#### 1001 species

**Figure 10.**
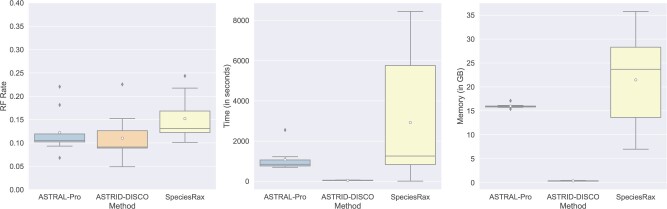
Experiment 2. Species tree error (RF error rates) and wall clock running time (s) on the data set containing 1001 species (1000 in-group species and one outgroup) and 50 gene trees estimated from 100bp alignments (44.4% MGTE). All the data sets have AD = 20%, a duplication rate of $5.0 \times 10^{-10}$, and an equal loss rate. CA-DISCO exceeded our 4-h time limit on all 10 of the replicates and is therefore omitted.

With 1001 species (1000 in-group species and one outgroup), we found that ASTRAL-Pro and ASTRID-DISCO gave the best accuracy, with ASTRID-DISCO utilizing significantly less memory and running time (Fig. 10). SpeciesRax was noticeably slower than ASTRAL-Pro and also less accurate. The peak memory usage is also higher for both ASTRAL-Pro and SpeciesRax than it is for ASTRID-DISCO. CA-DISCO failed to complete under our four-hour time constraints.

### Experiment 3

In Experiment 3, we examine the performance of ASTRAL-Pro, ASTRID-DISCO, and SpeciesRax on three empirical data sets: a 16-taxon fungi data set, an 83-taxon plant data set, and a 188-taxon vertebrate data set. To evaluate these estimated trees, we compute branch support on the estimated trees using ASTRAL’s posterior probability branch support technique (i.e., localPP) (Sayyari and Mirarab 2016). Full details and discussion are provided in Section S6 of the Supplementary material, and briefly summarized here.

Results on the 16-taxon fungal data set from Rasmussen and Kellis (2012) (Fig. S7 of the Supplementary material) show that all methods gave identical trees (the same tree returned by FastMulRFS on this data set in Molloy and Warnow (2020)) and in a reasonable amount of time. We compared it with the species tree given in Butler et al. (2009) and found that it disagreed on two branches. In contrast, the methods present different trees on the plant data set with 83 species and 9237 genes from Zhang et al. (2020) (Figs. S8–S10 and Table S8 of the Supplementary material) and the vertebrate data set with 188 species (Figs. S11–S13 and Table S9 of the Supplementary material). On the plant data set, all methods output a plausible species tree, disagreeing with the ASTRAL tree presented in the 1KP paper (Leebens-Mack et al. 2019) on only two to four branches, but in cases where the ground truth is not clear.

We ran ASTRAL-Pro, ASTRID-DISCO, and SpeciesRax on a vertebrate data set containing 188 species from Morel et al. (2021). ASTRAL-Pro and SpeciesRax both failed due to memory issues on Tallis (with 256 GB of available memory). ASTRID-DISCO finished and returned a tree that disagreed with the reference on five branches. Morel et al. (2021) reported in their analysis that ASTRAL-Pro and SpeciesRax disagreed on five branches as well.

**Figure 11.**
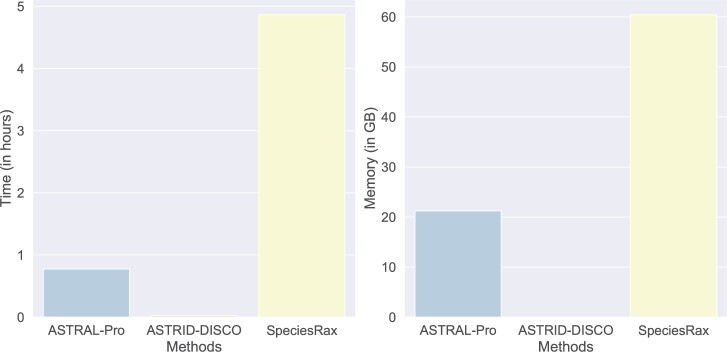
Experiment 3. Running time (h), and peak memory usage (GB) for the 1KP data set with 83 species and 9,237 gene family trees.

We also examined the running times and peak memory usage of the methods on the plant data set (Fig. 11). We see that ASTRID-DISCO requires less running time and memory compared to the other two methods (completing in about 90 s vs. approximately 45 min and almost 5 h for ASTRAL-Pro and SpeciesRax, respectively). Notably, we had an issue with SpeciesRax where it threw out of memory exceptions when we attempted to run it with parallelization (on a node with 256 GB of memory); this issue did not persist when we ran it serially, and thus we report those running times here.

### Failures to Complete

ASTRID-DISCO never failed on any data set, simulated or empirical. However, we observed several cases where ASTRAL-Pro, ASTRID-multi, or SpeciesRax failed to complete, either due to crashing or time constraints (Table 3). We examine these cases here.

**Table 3.** Failures to complete on simulated data. If model condition or method is not listed, it completed on all replicates. Timeouts are due to methods exceeding the Campus Cluster 4 h time limit. Memory means that the method’s memory requirements exceeded the amount of memory available (which was at least 64 GB).

Model Condition	Timeouts	Memory	Other	Total
ASTRAL-Pro				
(Fig. 3) 10,000 Gene Trees	10%	50%	0%	60%
(Fig. 4) Dup Rate $1 \times 10^{-9}$, loss 0	20%	0%	0%	20%
(Fig. 10) 1001 Species	0%	10%	0%	10%
ASTRAL-DISCO				
(Fig. 4) Dup Rate $1 \times 10^{-9}$, loss 0	0%	90%	0%	90%
(Fig. 4) Dup Rate $1 \times 10^{-9}$, loss 0.5	0%	90%	0%	90%
ASTRID-multi				
(Fig. 4) Dup Rate $1 \times 10^{-9}$, loss 0	10%	20%	0%	30%
(Fig. 4) Dup Rate $1 \times 10^{-9}$, loss 0.5	10%	0%	0%	10%
SpeciesRax				
(Fig. 4) Dup Rate $1 \times 10^{-9}$, loss 0	0%	10%	0%	10%
CA-DISCO (IQ-TREE)				
(Fig. 5) 1000 Gene Trees	100 %	0%	0%	100%
(Fig. 9) 1000 Gene Trees Missing Species	60%	0%	0%	60%
(Fig. 9) 10 Gene Trees Missing Species	0%	0%	10%	10%
(Fig. 10) 1001 Species	100%	0%	0%	100%

For our simulation study, we use the Campus Cluster, which is a heterogeneous computing cluster with a minimum memory of 64 GB and a time constraint of four hours. We found that ASTRAL-Pro failed several times due to memory given 10,000 gene trees and once with 1001 species; it also took too long (4 h) on the condition with the highest duplication rate and no loss (the condition yielding trees with the highest number of leaves). SpeciesRax almost always outputted a result (with the exception of one replicate with the highest duplication rate and no loss) as it computes a starting tree then proceeds to incrementally improve on this starting tree; any halts in its execution will just yield the tree in its current state instead of failing to give a result. However, there were several cases where SpeciesRax did not run to completion and failed to improve its starting MiniNJ tree. For example, in Experiment 1, there are several 1000-gene replicates on which SpeciesRax halts after a short period of time, returning a tree identical to the MiniNJ tree. For 10,000 genes this happens for all ten replicates. CA-DISCO (IQ-TREE) timed out on many replicates with large numbers of gene trees or species and also failed on one replicate 10 genes and missing species due to “problematic sequences.”

Both ASTRID-multi and ASTRAL-DISCO had issues on data sets with high duplication rates and low to moderate loss rates. ASTRAL-DISCO failed due to memory on nine out of ten replicates in two such cases. ASTRID-multi on the other hand fared a bit better, timing out on one replicate with no loss and one replicate with moderate loss and also running out of memory on two replicates with no loss.

When running our methods on empirical data sets, we used a node that has 256 GB of available memory and effectively no time constraints (i.e., the Tallis queue). Every method completed on the fungi data set (16 species) and also on the plants data set (83 species); however, on the vertebrates data set (188 species), only ASTRID-DISCO completed (SpeciesRax successfully computed its starting tree with MiniNJ, but halted soon afterward due to the memory constraints).

## Discussion

### Overview

DISCO is a new method for decomposing multicopy gene family trees into single copy trees. Its approach for decomposing the gene family trees relies explicitly on ASTRAL-Pro, which is used to “root and tag” the input gene family trees (and hence effectively predict orthology relationships) and then operates by splitting the tree into disjoint subtrees using the ASTRAL-Pro rooting and tagging. This decomposition produces subset trees that contain only orthologous genes when ASTRAL-Pro’s rooting and tagging is correctly performed, which enables us to prove that ASTRAL-DISCO is statistically consistent under GDL-only models. Moreover, our empirical study shows that even though DISCO does not have very high accuracy in its orthology determination, the species trees produced using DISCO in conjunction with several species tree estimation methods designed for single copy gene data sets are very accurate. Thus, DISCO is useful in phylogenomic species tree estimation when analyzing multicopy gene family data sets.

Although our study focused on the use of DISCO with ASTRAL, ASTRID, and concatenation analysis, it could be used with any species tree estimation method that is designed for single copy gene trees. For example, we studied concatenation analyses performed using maximum likelihood (under IQ-TREE) following DISCO decomposition, but other types of concatenation analysis could be performed, including distance-based methods. In addition, supertree methods, such as Matrix Representation with Parsimony, could be used to assemble a species tree from the decomposed single copy trees. Furthermore, under the assumption that the rooting and tagging is correctly performed, the decomposed single copy DISCO trees will only contain orthologous genes, and hence will be topologically identical to the species tree if GDL is the only cause for gene tree discord. Therefore, using DISCO with supertree methods such as MRP would also be statistically consistent under GDL-only models.

### ASTRID-DISCO and CA-DISCO

Our study specifically showed value for two applications of this method: ASTRID-DISCO and CA-DISCO. We found that ASTRID-DISCO was both accurate and extremely scalable. We found that it exhibited better accuracy than ASTRAL-DISCO, especially under conditions with few genes, where accuracy differences between methods are more pronounced. In our extensive simulation study, we did not find any condition where it performed worse than other methods (with the exception of CA-DISCO), and several where it performed better. For instance, it performed better than all other methods, including ASTRAL-Pro, under conditions with very few genes or very short sequence lengths (high MGTE). Its running times were far less than any other method with comparable accuracy, often ten times faster or more. Additionally, it is far more memory efficient, never exceeding peak memory of one gigabyte for any condition we measured. Finally, ASTRID-DISCO produced reasonable trees on the biological data sets, differing from those computed by ASTRAL-Pro or SpeciesRax only with respect to evolutionary relationships that are still under debate.

CA-DISCO typically tied for most accurate, but was strictly more accurate than the other tested methods (all of which are summary methods, and so operate by combining estimated gene trees) on conditions with high GTEE. In addition, when there was no heterogeneity due to ILS, CA-DISCO yielded better accuracy than all other methods; however, at high ILS it was less accurate than the best summary method (ASTRID-DISCO). A surprising finding is that CA-DISCO remained very accurate even when the concatenated alignment produced using DISCO was extremely gappy (typically above 90%, as shown in Table S4 of the Supplementary material). This robustness to missing data for IQ-TREE is noteworthy, given the general concerns raised about the impact of missing data on phylogenomic analyses (although see Molloy and Warnow (2018), which showed that several species tree estimation methods were able to recover highly accurate trees even given high levels of missing data). However, the robustness to missing data demonstrated by IQ-TREE may not hold for other maximum likelihood heuristics, and so future work is needed to explore this issue. Finally, we note that if missing data is of concern, the user can decide to limit the DISCO single copy gene trees to those that are sufficiently large, and hence reduce the degree of missing data in the input given to the species tree estimation method (whether concatenation analysis or some summary method).

We also saw that as the number of genes or the duplication rate increases, the size of the CA-DISCO data matrix (specifically in terms of the number of columns) increases. Even with relatively few sites per gene (as in our study), the total number of columns in the CA-DISCO matrix can be in the millions when the duplication rate is high enough (Table S4 of the Supplementary material). For instance, with a moderate duplication rate and 1000 genes (each gene alignment with length 100), we obtained concatenated alignments with 2.7 million columns on average (Table S4 of the Supplementary material). This presents significant runtime challenges to CA-DISCO and can make it infeasible to use under some conditions (especially when there is a large number of species). Moreover, it was under some of these challenging conditions where we were unable to estimate a tree under the 4-h time constraint in our experiment.

### ASTRAL-DISCO versus ASTRAL-Pro

Given that ASTRAL-DISCO uses DISCO to decompose the ASTRAL-Pro rooted-and-tagged gene family trees, it is interesting to compare ASTRAL-Pro and ASTRAL-DISCO. Note that while they are often similar in accuracy (and ASTRAL-DISCO is generally faster than ASTRAL-Pro), there are conditions with low numbers of genes where ASTRAL-Pro is more accurate (Fig. 3). Why is this?

Given a rooted and tagged gene tree, ASTRAL-Pro scores a quartet tree $q$ if this quartet is speciation-driven (meaning that it has four distinct species labels and the least common ancestor (LCA) of any three leaves is a speciation node). Since DISCO uses the rooting and tagging provided by ASTRAL-Pro, this implies that every quartet tree used by ASTRAL-DISCO is also considered by ASTRAL-Pro. However, in Figure S6 of the Supplementary material, we show an example of an input gene family tree on six species with one duplication event where ASTRAL-DISCO does not consider all of the quartet trees considered by ASTRAL-Pro. Hence, ASTRAL-Pro but not ASTRAL-DISCO makes full use of the speciation-driven quartet trees. This may explain why ASTRAL-Pro is more accurate than ASTRAL-DISCO when the number of genes is sufficiently small.

### ASTRID-Multi Running Time

A comparison between methods with respect to running time shows that ASTRID-multi is slower than ASTRID-DISCO and MiniNJ, the other two distance matrix methods we explore, when there are high rates of duplication and low rates of loss. Recall that ASTRID-multi is designed for multi-individual inputs, and that the matrix it computes has a row and column for each leaf in each gene family tree (which are interpreted as individuals). Hence, with high duplication rates and no loss, the number of leaves in the gene family trees is extremely large, leading to a large matrix. This problem is resolved by both MiniNJ and ASTRID-DISCO, as both their distance matrices scale only with the number of species.

### Causes of Missing Data

Analyses using DISCO decompositions tend to have substantial amounts of missing data: the individual single copy DISCO trees are in many cases much smaller than the gene family trees from which they are derived and the concatenation analyses are based on very gappy CA-DISCO alignments (Table S4 of the Supplementary material). Here we explore the causes for these high levels of missing data, focusing on gappiness in the CA-DISCO matrix (see Section S4 of the Supplementary material for further discussion).

Under all model conditions we explored, the CA-DISCO matrices are highly gapped (typically more than 93% gapped), as shown in Table S4 of the Supplementary material. Recall that the number of rows in the CA-DISCO matrix is the number of species and that each DISCO subset (i.e., leafset from a DISCO tree) contributes its associated set of columns in the matrix. Hence, the average size of the DISCO subsets (i.e., the average number of leaves in the DISCO trees), relative to the total number of species, determines the degree to which the CA-DISCO matrix is occupied by gaps. By Table S4 of the Supplementary material, the average size of the DISCO trees is typically between 4 and 7 for 101-species data sets (the one exception where the average is larger is the model condition with a low duplication rate, which is also the least gappy condition). We also note that the CA-DISCO matrix gappiness is not particularly large (compared to the other model conditions) for the Missing Data conditions (and correspondingly the average DISCO tree size is also not particularly small for that condition).

To understand why the typical DISCO tree is so small, we first consider the size of the gene family trees from which they are derived. As seen in Table S2 of the Supplementary material, gene family trees can have large numbers of leaves (averages as high as 3728 for the model condition with 101 species and high rates of duplication but no losses). Gene family trees also tend to have fewer than the full set of species, but a large reduction only appears under high rates of duplication and where loss also occurs (Table S3 of the Supplementary material). Furthermore, as noted earlier, the average DISCO tree size is not very affected by the Missing Data process implemented in Experiment 3.

Based on these trends, we conclude that the major cause for the CA-DISCO matrix to be so gappy is the decomposition strategy itself. This makes sense, when the DISCO decomposition strategy is considered. Recall that DISCO tries to produce at least one very large single copy tree, but this does not mean all (or even most) of its decomposed trees will be large. Also, when a duplication occurs close to the leaves, then unless a large number of losses also occurs (sufficient to remove at least one copy of each duplicated gene), the DISCO decomposition will by necessity produce a small tree. Furthermore, when used with concatenation, no DISCO trees are filtered out, and so DISCO has 100% coverage (i.e., every leaf in the input gene family tree is included in an output DISCO tree). This combination of seeking at least one very large single copy tree and also having 100% coverage results in a large number of DISCO trees (Table S5 of the Supplementary material), most of which are very small (Table S4 of the Supplementary material).

When using DISCO with summary methods such as ASTRAL or ASTRID that use single-copy unrooted gene tree topologies to estimate the species tree, we filter out all DISCO trees that have fewer than four species; in that case, the average size of the DISCO trees is much larger (approximately 50 species for the low duplication rate conditions, and decreasing to about 13 for the high duplication rate conditions, as seen in Table S5 of the Supplementary material). Thus, unfiltered DISCO decompositions produce small trees and result in CA-DISCO matrices that are very gappy; filtering the CA-DISCO analysis pipeline (by removing genes that are smaller than four species) would reduce gappiness but would still result in a gappy matrix.

In sum, by far the major cause for “missing data” in our study is the use of the DISCO decomposition, which produces large numbers of single copy trees, many of which are small. Filtering would increase the size of the trees and reduce gappiness, but the impact on accuracy resulting from filtering is unknown.

### DISCO Orthology

The set of leaves in each DISCO tree can be considered a set of putative orthologs, and the set of DISCO trees produced by decomposing a gene family tree defines disjoint orthogroups. This estimation of orthology relationships can then be compared to the true pairwise orthology relationships (known when performing a simulation) and evaluated for accuracy. We refer to the true relationships defined by the simulation as “actual orthologs” and “actual paralogs,” and then use these to specify whether DISCO’s orthology assessment is accurate (i.e., true positives are for pairs where DISCO predicts orthology and the pair are actual orthologs and true negatives are for pairs where DISCO predicts paralogy and the pair are actual paralogs) or inaccurate (i.e., false positives and false negatives, defined similarly) for each pair of gene copies. However, since this decomposition depends on ASTRAL-Pro’s rooting and tagging, to understand the orthology estimation accuracy obtained using DISCO, we consider the orthology accuracy for ASTRAL-Pro separately as well. We report both precision (i.e., TP/(TP+FP)) and recall (i.e., TP/(TP+FN)) for both ASTRAL-Pro and DISCO in Table S6 of the Supplementary material.

Given true gene trees, ASTRAL-Pro ranges from 0.35 to 1.0 for precision and from 0.65 to 0.92 for recall. The failure to achieve perfect precision and recall is due to both the existence of ILS in the gene trees and also to the rooting and tagging performed by ASTRAL-Pro since its optimization criterion (gene tree parsimony) may make mistakes. On estimated gene trees, ASTRAL-Pro orthology precision changes very little, but the recall drops (ranging now from 0.50 to 0.90). Thus, gene tree estimation error hardly impacts precision but substantially impacts recall.

Comparing DISCO to ASTRAL-Pro on true gene trees, we note that DISCO has better precision but lower recall than ASTRAL-Pro. For example, on true gene trees, DISCO precision values range from 0.45 to 1.0 (ASTRAL-Pro’s range from 0.35 to 1.0) and its recall values range from 0.33 to 0.86 (ASTRAL-Pro’s ranged from 0.65 to 0.92). On estimated gene trees a similar trend is seen, with DISCO precision improved but recall reduced, relative to ASTRAL-Pro. These changes are to some extent expected because orthology is not transitive, and DISCO can only recover some of the relationships because it partitions the leafset into disjoint sets. However, it is noteworthy that by decomposing gene family trees using ASTRAL-Pro’s rooting and tagging, DISCO retains most but not all of the accurate orthology relationships (thus reducing recall) and loses some of the incorrect orthology relationships (thus improving precision).

We now compare MI’s orthology accuracy to DISCO, as computed on estimated gene trees (Table S7 of the Supplementary material). MI has higher precision (0.76 compared to 0.53) but lower recall (0.32 compared to 0.41) than DISCO. Thus, MI misses more of the true orthologous relationships but the relationships it infers are more likely to be correct. This difference between MI and DISCO is explained in part by the trend for MI to make smaller trees and to cover less of the gene family tree leafset; by being more conservative in producing orthologous groups, MI makes fewer mistakes but also misses some of the true relationships.

Even so, the species trees produced using DISCO with different species tree estimation methods have higher accuracy than species trees estimated using MI decompositions, perhaps suggesting that DISCO benefits more from its larger coverage of the gene family trees and larger recall than it is hurt by the reduction in precision. These results also demonstrate the general robustness to errors in these orthology estimates (a trend that has been noted already in Yan et al. (2022)).

### Previous Studies

Five recent studies—Molloy and Warnow (2020), Zhang et al. (2020), Legried et al. (2021), (Willson et al., 2021), Yan et al. (2022), and Morel et al. (2021)—explored different multilocus species tree estimation methods on simulated data sets that evolved with GDL and potentially other sources of discord. Although the specific methods and model conditions varied between the studies, in most cases the trends in these studies are consistent with the trends that we also find.

The one study that reported different relative performance between methods than we find in this study is Morel et al. (2021), who found SpeciesRax and MiniNJ to be more accurate than ASTRAL-Pro under most conditions and only less accurate on a few conditions in which the differences were very small. Given that we found SpeciesRax and ASTRAL-Pro to be very close in accuracy, with no significant advantage to either method, we consider the relative performance reported in the two studies to be different enough to require some discussion.

The most likely explanation for the differences in trends between our study and Morel et al. (2021) is that the model conditions explored in the two studies are different. Specifically, most of the conditions explored in Morel et al. (2021) have only 25 species, whereas our study explored data sets with 101 species or 1001 species. Other studies (e.g., Nakhleh et al. 2001) have also shown that relative performance between methods can change with the number of species, and so this a distinct possibility. Although Morel et al. (2021) explored a range of model conditions, their default condition was very different from our default condition. Specifically, their default condition has high HGT and GDL but no ILS, uses only 100 genes, and has only 25 species; in contrast, our default model condition has ILS and GDL but no HGT, and 101 species. These differences may also have contributed to this relative performance advantage for SpeciesRax in their study. Significantly, the experiments they performed that did not have high HGT showed little difference between the two methods under most conditions, and no difference with 500 or more genes. Since ASTRAL-Pro performs its search within a constrained search space that is defined by the set of input gene family trees, this suggests that ASTRAL-Pro may not be as accurate as SpeciesRax when the number of genes is small. Overall, we conjecture that it is likely that SpeciesRax may have an advantage over ASTRAL-Pro for high HGT conditions and for conditions with no HGT but small numbers of genes and species.

A discussion of prior work examining concatenation in the presence of GDL is difficult since concatenation cannot be applied directly to multicopy gene families. However, comparisons of concatenation analyses using maximum likelihood to summary methods have been made for the specific case where ILS is the only cause for gene tree discordance. These studies have consistently shown that concatenation analyses can be more accurate than summary methods under some conditions (namely high gene tree estimation error and at most moderate ILS), while they can be less accurate under conditions with low gene tree estimation error and high ILS (see Kubatko and Degnan 2007; Leaché and Rannala 2011; Patel et al. 2013; DeGiorgio and Degnan 2014; Mirarab et al. 2016; Molloy and Warnow 2018 for an entry to this literature). The trends in our study for CA-DISCO in comparison to ASTRID-DISCO and ASTRAL-Pro are consistent with these prior studies and suggest the interesting potential for CA-DISCO to be a valuable method for species tree estimation under many conditions.

### Distance-Matrix Methods in the Presence of GDL

One consistent trend in our study is the good accuracy of summary methods that use distance matrices to estimate species trees in the presence of GDL. Overall, we have found ASTRID-DISCO to be the most accurate, while the relative accuracy of MiniNJ and ASTRID-multi depends on the model condition. Specifically, MiniNJ is less accurate than ASTRID-multi when given larger gene family trees or large numbers of genes. These two methods differ in two ways—how they calculate the distance matrix and how they estimate a tree from the calculated matrix (neighbor joining for MiniNJ and FastME for ASTRID). It is not clear which of these steps is most responsible for the difference in accuracy, but given the close similarity in accuracy for NJst (Liu and Yu 2011) and ASTRID (which also use neighbor joining and FastME, respectively), it is likely that the distance matrix calculation is the key reason for the differences between these methods.

Despite the very good accuracy of ASTRID-DISCO, it is not known whether it is statistically consistent under a GDL model. Furthermore, to date, no distance-matrix method has been proven statistically consistent under a model of GDL. There are two key factors in proving distance-based methods statistically consistent. First, the distance matrix must converge to an additive matrix as the number of gene trees increases; secondly, an appropriate method must be used to estimate the tree from the distance matrix. The second point is true for Neighbor Joining and FastME, the methods used by MiniNJ and ASTRID), but it is not known whether any of the suggested techniques for computing distance matrices will converge to an additive matrix under GDL models. While simulation studies are not sufficient to determine statistical consistency, we observe that as the number of gene trees increases, MiniNJ error seems to level off; in contrast, ASTRID-DISCO and ASTRID-multi error rates continue to decrease (Fig. 3). Future work is necessary to establish statistical consistency (or inconsistency) for these distance-matrix approaches.

## Conclusion

DISCO is a simple technique that opens up many new approaches to species tree inference in the context of gene duplication and loss. Here, we have shown two successful applications: ASTRID-DISCO and CA-DISCO. Using simulations, we showed that these two approaches work well in practice, with accuracy as good as or better than current leading methods, such as ASTRAL-Pro. Furthermore, ASTRID-DISCO performed well on three biological data sets, showing comparable accuracy to ASTRAL-Pro, while being much faster. Our study also suggests that CA-DISCO, applied using IQ-TREE, provides very good accuracy, comparable to or better than ASTRID-DISCO under most conditions (except for very high ILS), and so CA-DISCO should be used when it is feasible. However, CA-DISCO is more computationally intensive than the alternative methods, and ASTRID-DISCO is extremely fast. Hence, ASTRID-DISCO is a very fast and highly accurate method for species tree estimation that can handle GDL and ILS.

Much future work still needs to be done. One important direction is to establish the theoretical properties of these pipelines (e.g., statistical consistency and sample complexity), without making unnecessarily strong assumptions about correct rooting and tagging. For example, can statistical consistency be established when the probability of incorrect rooting and tagging is bounded? Future work should also explore the theoretical and empirical properties of CA-DISCO, ASTRID-DISCO, and other methods on datasets involving HGT, gene flow, and other sources of gene tree discord. While both ASTRID-DISCO and CA-DISCO performed well under the clade-based model of missing data, it would be interesting to see how well these methods perform in the context of other missing data models, such as the $M_{iid}$ model from Nute et al. (2018). It is also possible that CA-DISCO would yield results with even better accuracy when using RAxML (Stamatakis 2014) or perhaps a Bayesian tree estimation method, but this would require additional computational resources and so future studies should examine this. Finally, DISCO by design depends on ASTRAL-Pro for rooting and tagging the gene family trees; therefore, DISCO could potentially be improved by using techniques other than ASTRAL-Pro to root-and-tag the gene family trees.

We close with the observation that the good accuracy of DISCO pipelines suggests that other decomposition strategies that produce single copy gene trees might provide comparable or better accuracy, and potentially with lower computational cost. Furthermore, evaluating the impact on pipelines using DISCO that restrict the DISCO trees to those that are bigger than some threshold (e.g., 10% of the full set of species) could potentially improve accuracy and reduce running time. In general, future work into new decomposition strategies is desirable.

## Supplementary material

Datasets simulated for this study are available from the Illinois Databank at https://databank.illinois.edu/datasets/IDB-4050038. The DISCO algorithm is available in open-source form at: https://github.com/JSdoubleL/DISCO. DISCO has an external dependency on TreeSwift (v 1.1.14) (Moshiri, 2020).

## References

[B1] Allman E.S., Degnan J.H., Rhodes J.A. 2016. Species tree inference from gene splits by unrooted STAR methods. IEEE/ACM Trans. Comput. Biol. Bioinformatics 15:337–342. 10.1109/TCBB.2016.2604812 PMC5388605 28113601

[B2] Altenhoff A.M., Glover N.M., Dessimoz C. 2019. Inferring orthology and paralogy. In: Anisimova M., editor. Evolutionary genomics: statistical and computational methods. New York, NY: Springer New York. p. 149–175.

[B3] Arvestad L., Lagergren J., Sennblad B. 2009. The gene evolution model and computing its associated probabilities. J. ACM 56:1–44.

[B4] Ballesteros J.A., Hormiga G. 2016. A new orthology assessment method for phylogenomic data: unrooted phylogenetic orthology. Mol. Biol. Evol. 33:2117–2134. 27189539 10.1093/molbev/msw069

[B5] Bayzid M.S., Mirarab S., Warno T. 2013. Inferring optimal species trees under gene duplication and loss. Biocomputing. World Scientific. p. 250–261. 10.1142/9789814447973_0025 23424130

[B6] Boussau B., Szöllősi G.J., Duret L., Gouy M., Tannier E., Daubin V. 2013. Genome-scale coestimation of species and gene trees. Genome Res. 23:323–330. 23132911 10.1101/gr.141978.112 PMC3561873

[B7] Butler G., Rasmussen M. D., Lin M.F., Santos M.A., Sakthikumar S., Munro C.A., Rheinbay E., Grabherr M., Forche A., Reedy J.L., Agrafioti I., Arnaud M.B., Bates S., Brown A.J., Brunke S., Costanzo M.C., Fitzpatrick D.A., de Groot P.W., Harris D., Hoyer L.L., Hube B., Klis F.M., Kodira C., Lennard N., Logue M.E., Martin R., Neiman A.M., Nikolaou E., Quail M.A., Quinn J., Santos M.C., Schmitzberger F.F., Sherlock G., Shah P., Silverstein K.A., Skrzypek M.S., Soll D., Staggs R., Stansfield I., Stumpf M.P., Sudbery P.E., Srikantha T., Zeng Q., Berman J., Berriman M., Heitman J., Gow N.A., Lorenz M.C., Birren B.W., Kellis M., Cuomo C.A. 2009. Evolution of pathogenicity and sexual reproduction in eight candida genomes. Nature 459:657–662. 19465905 10.1038/nature08064 PMC2834264

[B8] Chaudhary R., Bansal M.S., Wehe A., Fernández-Baca D., Eulenstein O. 2010. iGTP: a software package for large-scale gene tree parsimony analysis. BMC Bioinformatics 11:1–7. 21092314 10.1186/1471-2105-11-574 PMC3002902

[B9] Chaudhary R., Boussau B., Burleigh J.G., Fernández-Baca D. 2015a. Assessing approaches for inferring species trees from multi-copy genes. Syst. Biol. 64:325–339. 25540456 10.1093/sysbio/syu128

[B10] Chaudhary R., Fernández-Baca D., Burleigh J.G. 2015b. MulRF: a software package for phylogenetic analysis using multi-copy gene trees. Bioinformatics 31:432–433. 25273112 10.1093/bioinformatics/btu648

[B11] Cheon S., Zhang J., Park C. 2020. Is phylotranscriptomics as reliable as phylogenomics? Mol. Biol. Evol. 37:3672–3683. 32658973 10.1093/molbev/msaa181 PMC7743905

[B12] Chifman J., Kubatko L. 2014. Quartet inference from SNP data under the coalescent model. Bioinformatics 30:3317–3324. 25104814 10.1093/bioinformatics/btu530 PMC4296144

[B13] De Oliveira Martins L., Mallo D., Posada D. 2016. A Bayesian supertree model for genome-wide species tree reconstruction. Syst. Biol. 65:397–416. 25281847 10.1093/sysbio/syu082 PMC4851173

[B14] DeGiorgio M., Degnan J.H. 2014. Robustness to divergence time underestimation when inferring species trees from estimated gene trees. Syst. Biol. 63:66–82. 23988674 10.1093/sysbio/syt059

[B15] Dunn C.W., Howison M., Zapata F. 2013. Agalma: an automated phylogenomics workflow. BMC Bioinformatics 14:1–9. 24252138 10.1186/1471-2105-14-330 PMC3840672

[B16] Emms D., Kelly S. 2018. STAG: species tree inference from all genes. BioRxiv 267914. doi: 10.1101/267914.

[B17] Federhen S. 2012. The NCBI taxonomy database. Nucleic Acids Res. 40:D136–D143. 22139910 10.1093/nar/gkr1178 PMC3245000

[B18] Fitch W.M. 2000. Homology: a personal view on some of the problems. Trends Genet. 16:227–231. 10782117 10.1016/s0168-9525(00)02005-9

[B19] Fletcher W., Yang Z. 2009. INDELible: a flexible simulator of biological sequence evolution. Mol. Biol. Evol. 26:1879–1888. 19423664 10.1093/molbev/msp098 PMC2712615

[B20] Hejnol A., Obst M., Stamatakis A., Ott M., Rouse G.W., Edgecombe G.D., Martinez P., Baguñà J., Bailly X., Jondelius U., Wiens M., Müller W.E.G., Seaver E., Wheeler W.C., Martindale M.Q., Giribet G., Dunn C.W. 2009. Assessing the root of bilaterian animals with scalable phylogenomic methods. Proc. R. Soc. B 276:4261–4270. 10.1098/rspb.2009.0896 PMC2817096 19759036

[B21] Heled J., Drummond A.J. 2009. Bayesian inference of species trees from multilocus data. Mol. Biol. Evol. 27:570–580. 19906793 10.1093/molbev/msp274 PMC2822290

[B22] Hudson R.R. 1983. Testing the constant-rate neutral allele model with protein sequence data. Evolution 203–217. 28568026 10.1111/j.1558-5646.1983.tb05528.x

[B23] Kingman J.F.C. 1982. The coalescent. Stoch. Process. Their Appl. 13:235–248.

[B24] Kocot K.M., Citarella M.R., Moroz L.L., Halanych K.M. 2013. PhyloTreePruner: a phylogenetic tree-based approach for selection of orthologous sequences for phylogenomics. Evol. Bioinformatics 9:EBO–S12813. 10.4137/EBO.S12813 PMC3825643 24250218

[B25] Kubatko L.S., Degnan J.H. 2007. Inconsistency of phylogenetic estimates from concatenated data under coalescence. Syst. Biol. 56:17–24. 17366134 10.1080/10635150601146041

[B26] Larget, B. R., Kotha S. K., Dewey C. N., and Ané C. 2010. BUCKy: gene tree/species tree reconciliation with Bayesian concordance analysis. Bioinformatics 26:2910–2911. 20861028 10.1093/bioinformatics/btq539

[B27] Leaché A.D., Rannala B. 2011. The accuracy of species tree estimation under simulation: a comparison of methods. Syst. Biol. 60:126–137. 21088009 10.1093/sysbio/syq073

[B28] Leebens-Mack J.H., Barker M.S., Carpenter E.J., Deyholos M.K., Gitzendanner M.A., Graham S.W., Grosse I., Li Z., Melkonian M., Mirarab S., Porsch M., Quint M., Rensing S.A., Soltis D.E., Soltis P.S., Stevenson D.W., Ullrich K.K., Wickett N.J., DeGironimo L., Edger P.P., Jordon-Thaden I.E., Joya S., Liu T., Melkonian B., Miles N.W., Pokorny L., Quigley C., Thomas P., Villarreal J.C., M.M. Augustin, M.D. Barrett, R.S. Baucom, D.J. Beerling, R.M. Benstein, E. Biffin, S.F. Brockington, D.O. Burge, J.N. Burris, K.P. Burris, V. Burtet-Sarramegna, A.L. Caicedo, S.B. Cannon, Z. Çebi, Y. Chang, C. Chater, J.M. Cheeseman, T. Chen, N.D. Clarke, H. Clayton, S. Covshoff, B.J. Crandall-Stotler, H. Cross, C.W. dePamphilis, J.P. Der, R. Determann, R.C. Dickson, V.S. Di Stilio, S. Ellis, E. Fast, N. Feja, K.J. Field, D.A. Filatov, P.M. Finnegan, S.K. Floyd, B. Fogliani, N. García, G. Gâteblé, G.T. Godden, F.Q.Y. Goh, S. Greiner, A. Harkess, J.M. Heaney, K.E. Helliwell, K. Heyduk, J.M. Hibberd, R.G.J. Hodel, P.M. Hollingsworth, M.T.J. Johnson, R. Jost, B. Joyce, M.V. Kapralov, E. Kazamia, E.A. Kellogg, M.A. Koch, M. Von Konrat, K. Könyves, T.M. Kutchan, V. Lam, A. Larsson, A.R. Leitch, R. Lentz, F.-W. Li, A.J. Lowe, M. Ludwig, P.S. Manos, E. Mavrodiev, M.K. McCormick, M. McKain, T. McLellan, J.R. McNeal, R.E. Miller, M.N. Nelson, Y. Peng, P. Ralph, D. Real, C.W. Riggins, M. Ruhsam, R.F. Sage, A.K. Sakai, M. Scascitella, E.E. Schilling, E.-M. Schlösser, H. Sederoff, S. Servick, E.B. Sessa, A.J. Shaw, S.W. Shaw, E.M. Sigel, C. Skema, A.G. Smith, A. Smithson, C.N. Stewart, J.R. Stinchcombe, P. Szövényi, J.A. Tate, H. Tiebel, D. Trapnell, M. Villegente, C.-N. Wang, S.G. Weller, M. Wenzel, S. Weststrand, J.H. Westwood, D.F. Whigham, S. Wu, A.S. Wulff, Y. Yang, D. Zhu, C. Zhuang, J. Zuidof, M.W. Chase, J.C. Pires, C.J. Rothfels, J. Yu, C. Chen, L. Chen, S. Cheng, J. Li, R. Li, X. Li, H. Lu, Y. Ou, X. Sun, X. Tan, J. Tang, Z. Tian, F. Wang, J. Wang, X. Wei, X. Xu, Z. Yan, F. Yang, X. Zhong, F. Zhou, Y. Zhu, Y. Zhang, S. Ayyampalayam, T.J. Barkman, N. Nguyen, N. Matasci, D.R. Nelson, E. Sayyari, E.K. Wafula, R.L. Walls, T. Warnow, H. An, N. Arrigo, A.E. Baniaga, S. Galuska, S.A. Jorgensen, T.I. Kidder, H. Kong, P. Lu-Irving, H.E. Marx, X. Qi, C.R. Reardon, B.L. Sutherland, G.P. Tiley, S.R. Welles, R. Yu, S. Zhan, L. Gramzow, G. Theißen, G.K.-S. Wong; and One Thousand Plant Transcriptome Initiative. 2019. One thousand plant transcriptomes and the phylogenomics of green plants. Nature 574: 679–685. 10.1038/s41586-019-1693-2 PMC6872490 31645766

[B29] Lefort V., Desper R., Gascuel O. 2015. FastME 2.0: a comprehensive, accurate, and fast distance-based phylogeny inference program. Mol. Biol. Evol. 32:2798–2800. 26130081 10.1093/molbev/msv150 PMC4576710

[B30] Legried B., Molloy E.K., Warnow T., Roch S. 2021. Polynomial-time statistical estimation of species trees under gene duplication and loss. J. Comput. Biol. 28:452–468. 33325781 10.1089/cmb.2020.0424

[B31] Liu L., Yu L. 2011. Estimating species trees from unrooted gene trees. Syst. Biol. 60:661–667. 21447481 10.1093/sysbio/syr027

[B32] Liu L., Yu L., Edwards S.V. 2010. A maximum pseudo-likelihood approach for estimating species trees under the coalescent model. BMC Evol. Biol. 10:1–18. 20937096 10.1186/1471-2148-10-302 PMC2976751

[B33] Mallo D., de Oliveira Martins L., Posada D. 2016. SimPhy: phylogenomic simulation of gene, locus, and species trees. Syst. Biol. 65:334–344. 26526427 10.1093/sysbio/syv082 PMC4748750

[B34] Markin A., Eulenstein O. 2021. Quartet-based inference is statistically consistent under the unified duplication-loss-coalescence model. Bioinformatics doi: 10.1093/bioinformatics/btab414. PMC9113308 34048529

[B35] Mirarab S., Bayzid M.S., Warnow T. 2016. Evaluating summary methods for multilocus species tree estimation in the presence of incomplete lineage sorting. Syst. Biol. 65:366–380. 25164915 10.1093/sysbio/syu063

[B36] Mirarab S., Reaz R., Bayzid M.S., Zimmermann T., Swenson M.S., Warnow T. 2014. ASTRAL: genome-scale coalescent-based species tree estimation. Bioinformatics 30:i541–i548. 25161245 10.1093/bioinformatics/btu462 PMC4147915

[B37] Molloy E.K., Warnow T. 2018. To include or not to include: the impact of gene filtering on species tree estimation methods. Syst. Biol. 67:285–303. 29029338 10.1093/sysbio/syx077

[B38] Molloy E.K., Warnow T. 2020. FastMulRFS: fast and accurate species tree estimation under generic gene duplication and loss models. Bioinformatics 36:i57–i65. 32657396 10.1093/bioinformatics/btaa444 PMC7355287

[B39] Morel B., Kozlov A.M., Stamatakis A., Szöllősi G.J. 2020. GeneRax: a tool for species-tree-aware maximum likelihood-based gene family tree inference under gene duplication, transfer, and loss. Mol. Biol. Evol. 37:2763–2774. 32502238 10.1093/molbev/msaa141 PMC8312565

[B40] Morel B., Schade P., Lutteropp S., Williams T.A., Szöllösi G.J., Stamatakis A. 2021. SpeciesRax: a tool for maximum likelihood species tree inference from gene family trees under duplication, transfer, and loss. bioRxiv doi: 10.1101/2021.03.29.437460. PMC8826479 35021210

[B41] Moshiri N. 2020. TreeSwift: a massively scalable Python tree package. SoftwareX 11:100436. 35903557 10.1016/j.softx.2020.100436 PMC9328415

[B42] Nakhleh L., Roshan U., St. John K., Sun J., Warnow T. 2001. Designing fast converging phylogenetic methods. Bioinformatics 17:S190–S198. 11473009 10.1093/bioinformatics/17.suppl_1.s190

[B43] Nguyen L.-T., Schmidt H.A., Von Haeseler A., Minh B.Q.. 2015. IQ-TREE: a fast and effective stochastic algorithm for estimating maximum-likelihood phylogenies. Mol. Biol. Evol. 32:268–274. 25371430 10.1093/molbev/msu300 PMC4271533

[B44] Nute M., Chou J., Molloy E.K., Warnow T. 2018. The performance of coalescent-based species tree estimation methods under models of missing data. BMC Genomics 19:1–22. 29745854 10.1186/s12864-018-4619-8 PMC5998899

[B45] Patel S., Kimball R.T., Braun E.L. 2013. Error in phylogenetic estimation for bushes in the tree of life. J. Phylogenet. Evol. Biol 1:2.

[B46] Price M.N., Dehal P.S., Arkin A.P. 2010. FastTree 2—approximately maximum-likelihood trees for large alignments. PLoS One 5:e9490. 20224823 10.1371/journal.pone.0009490 PMC2835736

[B47] Rabiee M., Sayyari E., Mirarab S. 2019. Multi-allele species reconstruction using ASTRAL. Mol. Phylogenet. Evol. 130:286–296. 30393186 10.1016/j.ympev.2018.10.033

[B48] Rasmussen M.D., Kellis M. 2012. Unified modeling of gene duplication, loss, and coalescence using a locus tree. Genome Res. 22:755–765. 22271778 10.1101/gr.123901.111 PMC3317157

[B49] Robinson D.F., Foulds L.R. 1981. Comparison of phylogenetic trees. Math. Biosci. 53:131–147.

[B50] Roch S., Warnow T. 2015. On the robustness to gene tree estimation error (or lack thereof) of coalescent-based species tree methods. Syst. Biol. 64:663–676. 25813358 10.1093/sysbio/syv016

[B51] Saitou N., Nei M. 1987. The neighbor-joining method: a new method for reconstructing phylogenetic trees. Mol. Biol. Evol. 4:406–425. 3447015 10.1093/oxfordjournals.molbev.a040454

[B52] Sayyari E., Mirarab S. 2016. Fast coalescent-based computation of local branch support from quartet frequencies. Mol. Biol. Evol. 33:1654–1668. 27189547 10.1093/molbev/msw079 PMC4915361

[B53] Smith M.L., Hahn M.W. 2021. New approaches for inferring phylogenies in the presence of paralogs. Trends Genet. 37:174–187. 32921510 10.1016/j.tig.2020.08.012

[B54] Stamatakis A. 2014. RAxML version 8: a tool for phylogenetic analysis and post-analysis of large phylogenies. Bioinformatics 30:1312–1313. 24451623 10.1093/bioinformatics/btu033 PMC3998144

[B55] Takahata N. 1989. Gene genealogy in three related populations: consistency probability between gene and population trees. Genetics 122:957–966. 2759432 10.1093/genetics/122.4.957 PMC1203770

[B56] Tavaré S. 1986. Some probabilistic and statistical problems in the analysis of DNA sequences. Lectures on Mathematics in the Life Sciences 17:57–86.

[B57] Thalén F. 2018. PhyloPyPruner: tree-based orthology inference for phylogenomics with new methods for identifying and excluding contamination. Lund University Student Papers. Http://lup.lub.lu.se/student-papers/record/8963554.

[B58] Thalén F. 2021. Website for PhyloPyPruner. https://gitlab.com/ fethalen/phylopypruner/. Last accessed July 15, 2021.

[B59] Vachaspati P., Warnow T. 2015. ASTRID: accurate species trees from internode distances. BMC Genomics 16:S3. 10.1186/1471-2164-16-S10-S3 PMC4602181 26449326

[B60] Vachaspati P., Warnow T. 2017. FastRFS: fast and accurate Robinson–Foulds Supertrees using constrained exact optimization. Bioinformatics 33:631–639. 27663499 10.1093/bioinformatics/btw600 PMC5870905

[B61] Wehe, A., Bansal M. S., Burleigh J. G., Eulenstein O. 2008. DupTree: a program for large-scale phylogenetic analyses using gene tree parsimony. Bioinformatics 24:1540–1541. 18474508 10.1093/bioinformatics/btn230

[B62] Willson J., Roddur M. S., and Warnow T. 2021. Comparing methods for species tree estimation with gene duplication and loss. In: International Conference on Algorithms for Computational Biology. Springer. p. 106–117.

[B63] Yan Z., Smith M.L., Du P., Hahn M.W., Nakhleh L. 2022. Species tree inference methods intended to deal with Incomplete Lineage Sorting are robust to the presence of paralogs. Syst. Biol. syab056. 71:367–381. 10.1093/sysbio/syab056 PMC8978208 34245291

[B64] Yang Y., Smith S.A. 2014. Orthology inference in nonmodel organisms using transcriptomes and low-coverage genomes: improving accuracy and matrix occupancy for phylogenomics. Mol. Biol. Evol. 31:3081–3092. 25158799 10.1093/molbev/msu245 PMC4209138

[B65] Zhang C., Scornavacca C., Molloy E.K., Mirarab S. 2020. ASTRAL-Pro: quartet-based species-tree inference despite paralogy. Mol. Biol. Evol. 37:3292–3307. 32886770 10.1093/molbev/msaa139 PMC7751180

