# Correction to: DISCO: Species Tree Inference using Multicopy Gene Family Tree Decomposition

**DOI:** 10.1093/sysbio/syad003

**Published:** 2023-03-11

This is a correction to: James Willson, Mrinmoy Saha Roddur, Baqiao Liu, Paul Zaharias, Tandy Warnow, DISCO: Species Tree Inference using Multicopy Gene Family Tree Decomposition, *Systematic Biology*, Volume 71, Issue 3, May 2022, Pages 610–629, https://doi.org/10.1093/sysbio/syab070

In the originally published version of this manuscript the supplementary material file providing more information on the study was inadvertently omitted. This error has been corrected online.

